# Synthesis of Microspherical LiFePO_4_-Carbon Composites for Lithium-Ion Batteries

**DOI:** 10.3390/nano3030443

**Published:** 2013-07-22

**Authors:** Linghui Yu, Dandan Cai, Haihui Wang, Maria-Magdalena Titirici

**Affiliations:** 1 Max-Planck Institute for Colloids and Interfaces, Am Muehlenberg 1, Golm/Potsdam 14424, Germany; E-Mail: linghui.yu@mpikg.mpg.de; 2 School of Chemistry and Chemical Engineering, South China University of Technology, Wushan Road 381, Guangzhou 510640, China; E-Mails: cai.dd@mail.scut.edu.cn (D.C.); hhwang@scut.edu.cn (H.W.); 3 School of Engineering and Materials Science, Queen Mary University of London, Mile End Road, London E14NS, UK

**Keywords:** energy storage, nanomaterials, lithium-ion batteries, LiFePO_4_

## Abstract

This paper reports an “all in one” procedure to produce mesoporous, micro-spherical LiFePO_4_ composed of agglomerated crystalline nanoparticles. Each nanoparticle is individually coated with a thin glucose-derived carbon layer. The main advantage of the as-synthesized materials is their good performance at high charge-discharge rates. The nanoparticles and the mesoporosity guarantee a short bulk diffusion distance for both lithium ions and electrons, as well as additional active sites for the charge transfer reactions. At the same time, the thin interconnected carbon coating provides a conductive framework capable of delivering electrons to the nanostructured LiFePO_4_.

## 1. Introduction

LiFePO_4_ is one of the most promising cathode materials for the next generation of lithium ion batteries used in vehicles, due to its high theoretical capacity (170 mAh g^−1^), low cost, safety and environmental friendliness [1,2,3,4,5,6]. The key limitation of this material has been its inherent low electronic conductivity (10^−9^–10^−10^ S cm^−1^ at room temperature) [7,8,9,10]. Since it was first reported by Goodenough *et al*. in 1997 [1], tremendous strategies have been used to improve its performance as a cathode material for lithium ion batteries. These involved mainly coatings with conductive carbon [11,12,13,14] or doping with supervalent metals [10,15,16].

Carbon coating is the most widely used and straightforward technology to improve the conductivity of LiFePO_4_ materials. Recently reported nanostructured LiFePO_4_/C composites have demonstrated that this material can reach extremely high power [17,18,19]. However, homogenous and uniform carbon coating with a favourable structure remains still a challenge.

After more than 10 years of dedicated research, it is now clear that the prerequisite for a high-performance LiFePO_4_/C composite should be a nanosized material homogeneously coated with a thin layer of carbon. The role of carbon is to improve the conductivity of LiFePO_4_, while it should not block the lithium ion transfer between LiFePO_4_ and electrolyte. Nanosized particles have shorter diffusion paths for lithium ions and electrons as compared with bulk materials, which is beneficial for a fast charge/discharge capability. Such a quality is crucial for automotive applications. However, for practical applications, nanoparticles have a lower density than microparticles, resulting in a lower energy density. In addition, a material with good fluidity, such as spherical particles, is highly desired in industry, as it can be densely packed and, therefore, is favourable for electrode processing [18,20,21,22,23].

Taking into consideration all the previously mentioned aspects, spherical LiFePO_4_ of micrometre size made out of aggregated nanoparticles and additionally coated with a thin carbon layer should represent an ideal morphology for high-performance LiFePO_4_ cathode materials.

Hydrothermal synthesis represents a quick and easily scalable method to prepare various products (inorganic nanoparticles [24], polymers [25] and carbons [26,27]) with controlled morphology. To date, various morphologies for LiFePO_4_ have been already prepared by the hydrothermal method [21,28,29,30,31,32,33,34,35]. Here, a hydrothermal process is developed to synthesize in one step mesoporous LiFePO_4_ microspheres composed of nanoparticles, which are coated with a very thin and interconnected carbon layer. The thin hydrothermal carbon layer is produced simultaneously with the formation of LiFePO_4_ materials employing glucose, a biomass-derived precursor derived from cellulose hydrolysis. It provides a highly conductive framework to deliver electrons to nano LiFePO_4_ particles. Although there is still room for optimization in order to reach the theoretical capacity, the as-prepared LiFePO_4_ material with such distinct morphology exhibits an enhanced rate capability, especially when considering the low capacity achieved at a low current rate.

## 2. Results and Discussion

**Figure 1:**
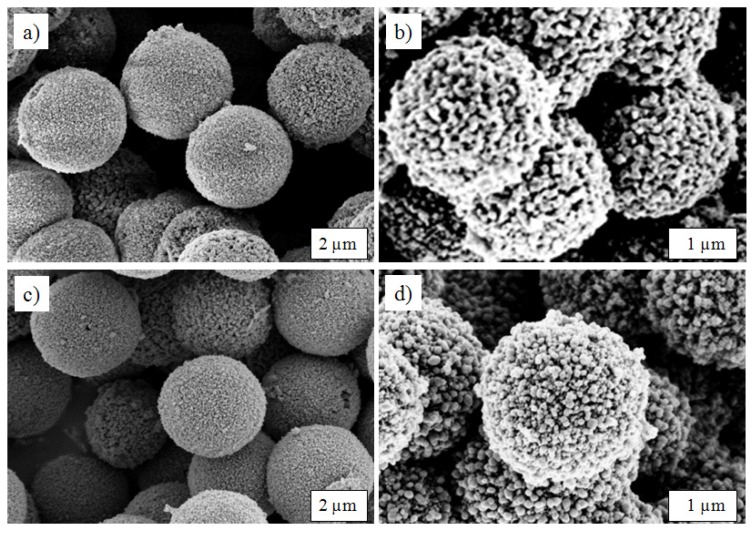
SEM images of LiFePO_4_/C spheres before (**a** and **b**) and after (**c** and **d**) calcination. (**a**) LFP180-2; (**b**) LFP250-1; (**c**) LFP 180-2-700; and (**d**) LFP250-1-700.

The SEM images (Figure 1a,b) show the monodispersed microspheres obtained after hydrothermal treatment. The micrometre-sized particles are made out of individual nanoparticles with diameters of 20–100 nm, depending on the synthesis conditions. The morphology did not change upon additional calcination at 700 °C, as shown in Figure 1c,d, as well as in Figures S1 and S2. With the microsized, spherical morphology, the materials own a high tap density, ~1.1 g cm^−3^ for LFP250-1-700, while a value of less than 1.0 g cm^−3^ is common for other LiFePO_4_/C composites [28].

**Figure 2:**
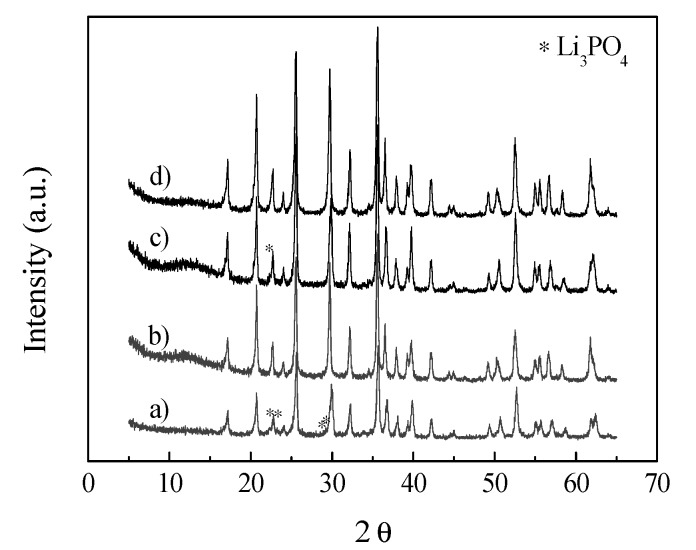
XRD patterns of the materials before (**a** and **b**) and after (**c** and **d**) calcination. (**a**) LFP-180; (**b**) LFP-180-700; (**c**) LFP-250; and (**d**) LFP-250-700.

The XRD patterns of the samples before and after calcination are shown in Figure 2. For the samples before calcination, besides the main diffraction peaks of an orthorhombic olivine-type LiFePO_4_, impurity Li_3_PO_4_ peaks are observed. Impurities were widely existent for LiFePO_4_ samples prepared by the hydrothermal method, as reported by many researchers [32,36,37], which might be due to the synthesis conditions, as well as the presence of the Fe(III). Note that the Fe(II) raw material can be easily oxidized with the presence of water and O_2_. However, after further solid-state reaction at 700 °C, all the peaks of the two samples can be indexed into an orthorhombic olivine-type LiFePO_4_ with a space group of Pnma. The carbon contents of the samples after calcination were determined by elemental analysis to be 3.2 wt.% and 2.8 wt.% for LFP180-2-700 and LFP250-1-700, respectively.

**Figure 3:**
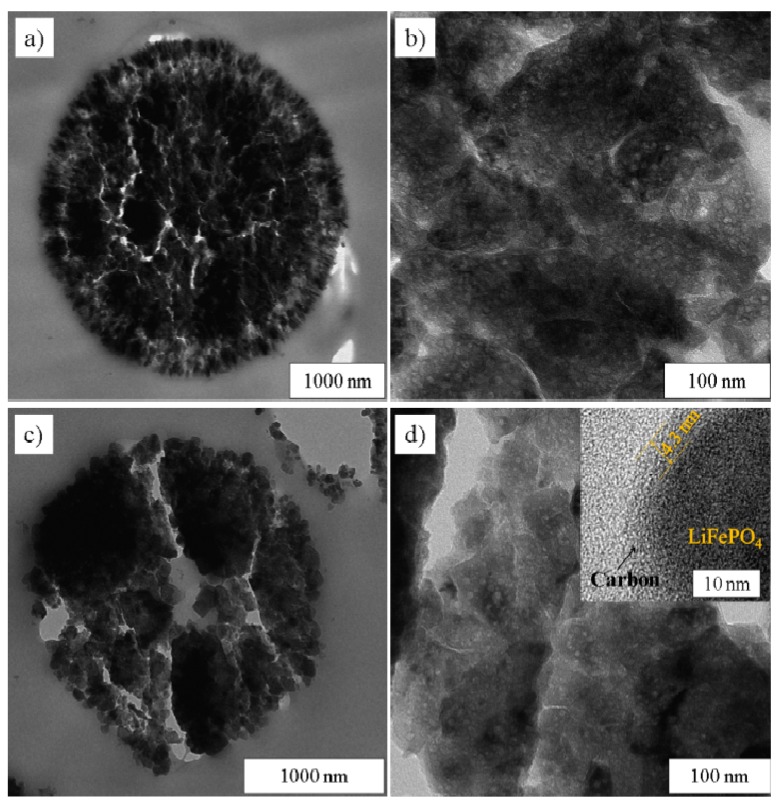
TEM images of ultramicrotome cross-sections of LiFePO_4_/C spheres. (**a**) and (**b**) LFP180-2-700; (**c**) and (**d**) LFP250-1-700; (**b**) and (**d**) are magnified images from inner parts. The insert in (**d**) is a high resolution tunnelling electron microscopy (HRTEM) image of LFP250-1-700.

Ultramicrotome cross-sections of LiFePO_4_/C spheres were examined by TEM in order to get better insights into their inner structure. Figure 3a,c show that the particles located at the surface are nanosized, about 20–100 nm, in agreement with the SEM images. The particles located in the interior of the microspheres seem to be larger, with sizes up to several hundreds of nanometres. However, the magnified images (Figure 3b,d) reveal that these big particles are actually also made up of small aggregated nanoparticles. From these TEM images, mesopores can be clearly observed, proving the mesoporosity of the materials. A carbon layer of ~4.3 nm can be observed in the high resolution tunnelling electron microscopy (HRTEM) picture (the insert in Figure 3d), indicating a thin layer of carbon coating on the LiFePO_4_ nanoparticles, which was further confirmed by investigation on the pure carbon after removing the LiFePO_4_ with HCl, as shown later.

**Figure 4:**
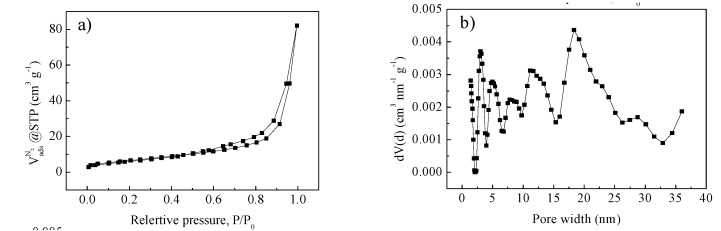
N_2_ adsorption data at 77.4 K. (**a**) N_2_ adsorption isotherm and (**b**) pore size distribution of LFP250-1-700.

N_2_ adsorption measurement was performed to further determine the porous structure. As shown in Figure 4a, a type IV isotherm with an H3 hysteresis loop can be observed for LFP250-1-700, confirming its mesoporosity. Pore size distribution (PSD, Figure 4b) shows a hierarchical mesoporosity with pore diameters in a wide range. The Brunauer-Emmett-Teller (BET) surface area and pore volume are 24.1 m^2^ g^−1^ and 0.076 cm^3^ g^−1^, respectively, which are low, due to the high density of these types of materials.

**Figure 5:**
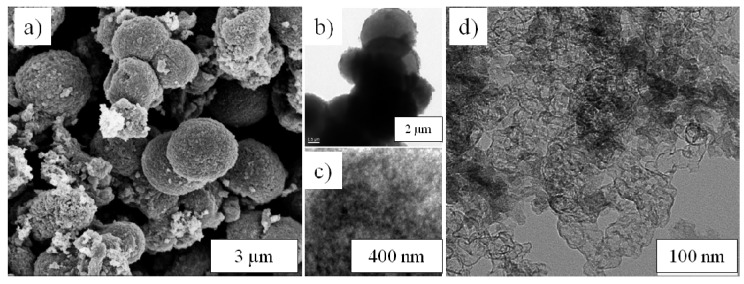
(**a**) SEM and (**b**–**d**) TEM images of the carbon coating from LFP250-1-700 after removing LiFePO_4_ by HCl (37%); (**c**) magnified TEM image; (**d**) TEM image of a broken part of the pure carbon.

In order to better understand the carbon coating, LFP250-1-700 was washed with HCl (37%) solution to remove the LiFePO_4_. The XRD pattern (Figure S3) of the resultant sample, with no peak corresponding to LiFePO_4_, confirms the removal of LiFePO_4_. SEM and TEM measurements were also performed on the pure carbon. As shown in Figure 5, both of the SEM and TEM images show very interesting morphology. The SEM image (Figure 5a) shows that, after removing the LiFePO_4_, the spherical morphology remained, although some degree of shrinkage occurred, and some spheres were cracked. The TEM image shown in Figure 5b confirms the spherical aspect. The magnified TEM image (Figure 5c) shows its interconnected nanostructure, which supports the spherical structure, as well as provides an interconnected conductive framework to deliver electrons to nano LiFePO_4_, which should be greatly beneficial for its high rate performance. It also shows that the pure carbon is mesoporous. The mesopores formed probably because of the removal of the LiFePO_4_. N_2_ adsorption also confirms its mesoporosity (Figure S4). The BET surface area and pore volume are 929.4 m^2^ g^−1^ and 1.22 cm^3^ g^−1^. Moreover, the TEM image (Figure 5d) from a broken part of the pure carbon shows the structure more clearly, namely, very thin hollow shells with an interconnected structure, in agreement with the observation of a very thin layer of carbon coating on the HRTEM image (the insert in Figure 3d).

**Figure 6:**
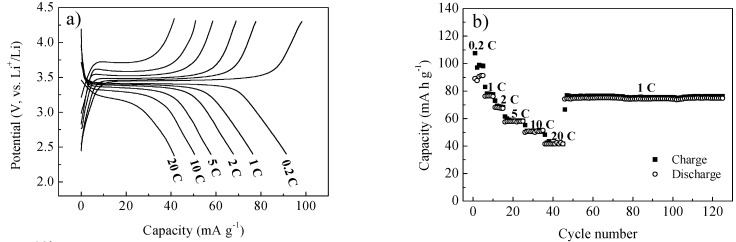
Electrochemical properties of LFP250-1-700. (**a**) Galvanostatic charge/discharge curves at different rates; and (**b**) corresponding cycling performance. 1 C = 170 mA g^−1^.

After the characterization of both the composite and the carbon coating, we have analysed the Li storage properties of such materials. Figure 6a shows the galvanostatic charge/discharge curves of LFP250-1-700. The sample has a capacity of ~90 mAh g^−1^ at a rate of 0.2 C, which is only a little higher than half of the theoretical capacity. However, the capacity retention with increasing the current rate is excellent. At a rate of 20 C (3400 mA g^−1^), it can still deliver a capacity of 42 mAh g^−1^. At such a high rate, it also shows modest polarization with a clear voltage plateau above 3 V, indicating the excellent kinetics [38]. This excellent capacity retention with the rate increasing could be attributed to the special structure of the material. Firstly, the interconnected, thin carbon coating provides a highly conductive framework removing the main restriction of the low conductivity of LiFePO_4_. Secondly, the nanosized particles, which aggregate into microspheres, and the mesoporosity of the material guarantee a short lithium diffusion distance in the bulk. The material also has stable cycling performance, as shown in Figure 6b. It was cycled at a rate of 1 C from the 46th cycle, and the capacity was always maintained at ~75 mAh g^−1^ during the next 80 cycles.

The performance of LiFePO_4_ is usually restricted by slow kinetics. However, here, the LiFePO_4_ spheres can provide fast kinetics, but a low capacity at a low rate. This phenomenon is probably associated with the existence of the impurity in the sample after hydrothermal reaction, as indicated by the XRD patterns. Further studies to clarify the small capacity at low rates are still needed at this point.

## 3. Experimental Section

### 3.1. Chemicals

D-(+)-glucose (C_6_H_12_O_6_ H_2_O) was purchased from Roth Chemicals. Iron(II) chloride tetrahydrate (FeCl_2_ 4H_2_O), ammonium phosphate dibasic [(NH_4_)_2_HPO_4_] and lithium hydroxide monohydrate (LiOH H_2_O) were purchased from Sigma-Aldrich (Taufkirchen, Germany).

### 3.2. Synthesis of LiFePO_4_/C Microspheres

Three micromoles of FeCl_2_ 4H_2_O were dissolved in 6 mL of 30 wt/v aqueous glucose (C_6_H_12_O_6_ H_2_O) solution. Nine millilitres of LiOH solution (1 mol L^−1^) and 3 mL of (NH_4_)_2_HPO_4_ (1 mol L^−1^) were shortly added to this mixture. After stirring for 5 min, the mixture was sealed into a Teflon-lined autoclave (Parr Instruments, Frankfurt, Germany) and kept in a preheated oven for 2 h (180 °C) or 1 h (250 °C). The products are denoted as LFP180-2 and LFP250-1, correspondingly. After cooling down in a water bath at ambient temperature, the precipitate was collected by centrifugation, washed with water and ethanol several times and, finally, dried in a vacuum at 80 °C. The dried powder was calcined at 700 °C under N_2_ for 8 h to obtain the final LiFePO_4_/C conductive spheres. The final products are denoted as LFP180-2-700 and LFP250-1-700, corresponding to their hydrothermal temperature and time.

### 3.3. Characterization

Scanning electron microscopy (SEM) images were collected on a Gemini Leo-1550 instrument (Carl Zeiss, Oberkochen, Germany). X-ray powder diffraction (XRD) analyses of the samples were carried out with a Bruker D8 Advance diffractometer (Bruker AXS GmbH, Karlsruhe, Germany) using CuKa radiation. Transmission electron microscopy (TEM) was performed on a Zeiss EM 912 instrument (Carl Zeiss, Oberkochen, Germany) equipped with a CCD camera and a filament of LaB6 under a 120 kV tension. High resolution tunnelling electron microscopy (HRTEM) was carried out using a Hitachi HF3300 cold-field emission TEM/STEM instrument (Hitachi High-Technologies Co., Tokyo, Japan) at 300 kV. Elemental composition was determined using a Vario El elemental analyser (Elementar Analysensysteme GmbH, Hanau). N_2_ adsorption analysis were performed using a QUADRASORB SI/MP (Quantachrome Instruments, Boynton Beach, FL, USA) at 77.4 K. Prior to measurement, the samples were degassed at 150 °C for 20 h. Brunauer-Emmett-Teller (BET) and non-linear density functional theory (NLDFT) methods were used for the surface area and pore size distribution (PSD) determination.

### 3.4. Electrochemical Measurements

The working electrode was made by spreading 80% active materials (LiFePO_4_/C), 12% carbon black and 8% poly(vinylidene difluoride) (PVDF) onto an aluminium current collector and dried in a vacuum oven at 120 °C overnight. 2032 coin cells were used as testing batteries and assembled in an argon-filled dry box. Lithium metal was used as a counter electrode. Celgard 2325 was used as a separator. The electrolyte was 1 M LiPF_6_ in ethylene carbonate/dimethyl carbonate (1/1 by volume). Galvanostatic charge and discharge (2.4–4.3 V) tests were performed on battery testing equipment (Neware Electronic Co., Shenzhen, China) at different current rates (1C = 170 mA g^−1^) under ambient temperature.

## 4. Conclusions

Mesoporous LiFePO_4_/C microspheres were prepared by a facile “all in one” hydrothermal method. These microspheres are composed of nanosized particles coated with a very thin and interconnected carbon layer. The interconnected carbon structure provides a conductive framework capable of delivering electrons to each nano LiFePO_4_ particle. The nanoparticles and the mesoporosity provide a large number of active sites for charge transfer reaction and a short bulk diffusion distance for both lithium ions and electrons. With such a structure, the prepared material exhibits excellent capacity retention with the increase of current rate. The low capacity at a low rate could be probably associated with the existence of the impurities in the sample after hydrothermal reaction, which needs to be further studied.

## Supplementary Materials

Supplementary File 1
