# Supplementary material for: Synthesis of Microspherical LiFePO4-Carbon Composites for Lithium-Ion Batteries
Source: Nanomaterials (Basel). 2013 Jul 22;3(3):443–52. doi: 10.3390/nano3030443 (PMC5304656; doi:10.3390/nano3030443)
Supplement: Supplementary File 1 [file nanomaterials-03-00443-s001.doc]

Supplementary Information

**Figure S1.** Low magnification SEM image of LFP 180-2-700.


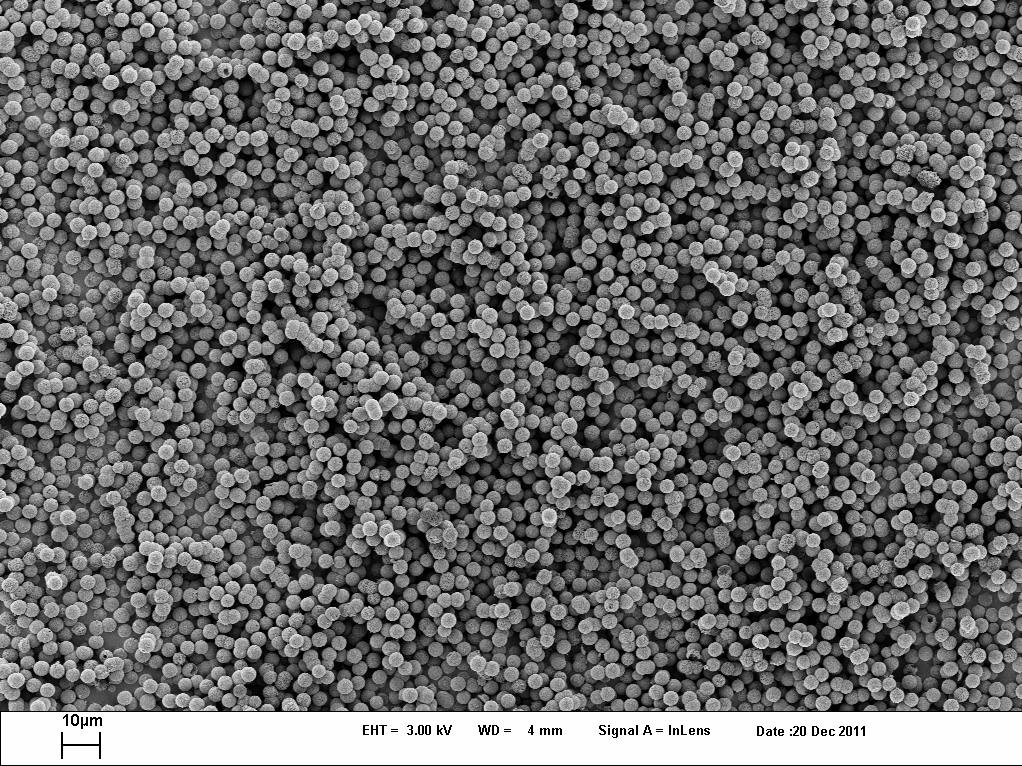


**Figure S2.** Low magnification SEM image of LFP 250-1-700.


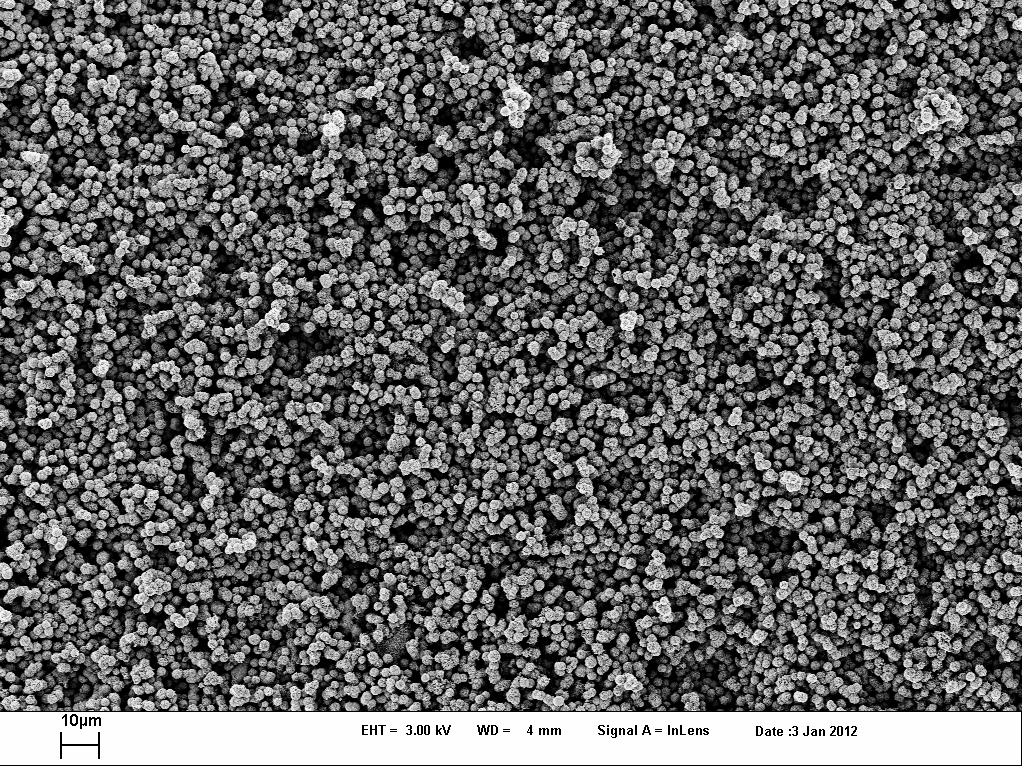


**Figure S3.** XRD pattern of the pure carbon after removing LiFePO_4_ with HCl.

**Figure S4.** (**a**) N_2_ adsorption isotherm and (**b**) PSD the pure carbon from LFP250-1-700 after removing LiFePO_4_ with HCl.

© 2013 by the authors; licensee MDPI, Basel, Switzerland. This article is an open access article distributed under the terms and conditions of the Creative Commons Attribution license (http://creativecommons.org/licenses/by/3.0/).
